# Estrogen Regulates Hepcidin Expression via GPR30-BMP6-Dependent Signaling in Hepatocytes

**DOI:** 10.1371/journal.pone.0040465

**Published:** 2012-07-11

**Authors:** Yasumasa Ikeda, Soichiro Tajima, Yuki Izawa-Ishizawa, Yoshitaka Kihira, Keisuke Ishizawa, Shuhei Tomita, Koichiro Tsuchiya, Toshiaki Tamaki

**Affiliations:** 1 Department of Pharmacology, Institute of Health Biosciences, The University of Tokushima Graduate School, Tokushima, Japan; 2 Department of Medical Pharmacology, Institute of Health Biosciences, The University of Tokushima Graduate School, Tokushima, Japan; University of Valencia, Spain

## Abstract

Hepcidin, a liver-derived iron regulatory protein, plays a crucial role in iron metabolism. It is known that gender differences exist with respect to iron storage in the body; however, the effects of sex steroid hormones on iron metabolism are not completely understood. We focused on the effects of the female sex hormone estrogen on hepcidin expression. First, ovariectomized (OVX) and sham-operated mice were employed to investigate the effects of estrogen on hepcidin expression in an *in vivo* study. Hepcidin expression was decreased in the livers of OVX mice compared to the sham-operated mice. In OVX mice, bone morphologic protein-6 (BMP6), a regulator of hepcidin, was also found to be downregulated in the liver, whereas ferroportin (FPN), an iron export protein, was upregulated in the duodenum. Both serum and liver iron concentrations were elevated in OVX mice relative to their concentrations in sham-operated mice. In *in vitro* studies, 17β-estradiol (E_2_) increased the mRNA expression of hepcidin in HepG2 cells in a concentration-dependent manner. E_2_-induced hepatic hepcidin upregulation was not inhibited by ICI 182720, an inhibitor of the estrogen receptor; instead, hepcidin expression was increased by ICI 182720. E_2_ and ICI 182720 exhibit agonist actions with G-protein coupled receptor 30 (GPR30), the 7-transmembrane estrogen receptor. G1, a GPR30 agonist, upregulated hepcidin expression, and GPR30 siRNA treatment abolished E_2_-induced hepcidin expression. BMP6 expression induced by E_2_ was abolished by GPR30 silencing. Finally, both E_2_ and G1 supplementation restored reduced hepatic hepcidin and BMP6 expression and reversed the augmentation of duodenal FPN expression in the OVX mice. In contrast, serum hepcidin was elevated in OVX mice, which was reversed in these mice with E_2_ and G1. Thus, estrogen is involved in hepcidin expression via a GPR30-BMP6-dependent mechanism, providing new insight into the role of estrogen in iron metabolism.

## Introduction

Iron is an essential trace element that is necessary for all living cells and organisms. Approximately 3–5 g of iron is stored by healthy adults, and the amount of stored iron varies during the lifetime. It is well known that gender differences exist with respect to iron storage. Women exhibit low levels of iron storage from adolescence to menopause. In perimenopausal women, iron levels in the body increase to levels similar to those in men of the same age [1]. Lower iron storage levels in women is simply believed to be associated with blood loss during menstruation, which explains the increase in iron storage by women during perimenopause [2].

Menopause is detrimental for middle-aged and older women because of various so-called menopausal disorders. Menopause is generally considered to occur because of a deficiency in the female sex steroid hormone estrogen. In women, estrogen levels increase around adolescence and remain constant until perimenopause, when its levels decrease because of the cessation of ovarian function in the postmenopausal period. In terms of the relationship between iron and estrogen, it has been observed that there is a coincidental and inverse correlation between estrogen levels and iron storage during perimenopause [2]. Recently, several studies have demonstrated that estrogen exerts various effects on multiple organs, including both classical target organs and non-classical target organs [3]. We hypothesized that the reduction of estrogen levels due to menopause is responsible for increased iron accumulation due to changes in iron regulation. However, the effects of estrogen on iron regulation remain unknown.

Hepcidin is a secreted protein derived from hepatocytes, and it has been identified as an important regulator of iron metabolism [4]. Indeed, hepcidin has been demonstrated to regulate iron absorption in the duodenum through internalization and degradation of ferroportin (FPN), an exporter of iron [5]. In the present study, we demonstrate a new effect of estrogen on iron metabolism. Estrogen regulates hepcidin expression via G-coupled protein 30 (GPR30)–bone morphologic protein 6 (BMP6)-dependent signaling, indicating that estrogen actually decreases iron absorption in the intestine.

## Results

### Effect of Estrogen on Hepcidin Expression and Iron Absorption in vivo

**Figure 1:**
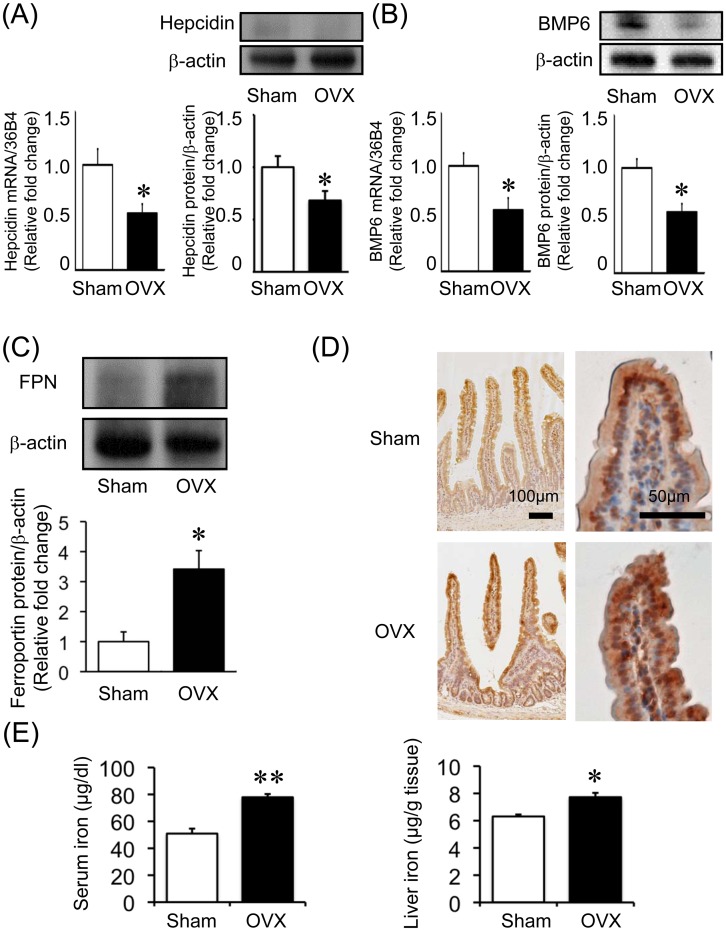
Effect of estrogen deprivation via an ovariectomy on hepcidin expression and iron absorption *in vivo*. The expression of hepcidin (A) and bone morphological protein (BMP6) (B) was reduced in the livers of the OVX mice. The expression of hepcidin and BMP6 in liver tissues at 3 months after the sham operation or ovariectomy was analyzed. The expression values are expressed as mean ± SEM; *P<0.05, **P<0.01, *n* = 6–10 in each group. (C) The expression of ferroportin (FPN) was augmented in the duodena of the OVX mice. The protein expression of FPN in duodenal tissues at 3 months after the sham operation or ovariectomy was analyzed. Values are expressed as mean ± SEM; *P<0.05, **P<0.01, *n* = 6 in each group. (D) Immunohistochemical analysis of FPN expression in the duodena of sham-operated mice (upper) and OVX mice (lower). (E) Serum (µg⋅dl^−1^ ) and hepatic (ng⋅g^−1^ ) iron concentrations in sham-operated and OVX mice. Values are expressed as mean ± SEM; *P<0.05, **P<0.01, n = 8–12 in each group.

To elucidate the effect of estrogen on iron metabolism *in vivo*, we compared hepcidin and iron levels between OVX and sham-operated mice. Both mRNA and protein expression of hepcidin was significantly reduced in the livers of OVX mice as compared to the expression in the livers of sham-operated mice. Furthermore, the mRNA and protein levels of BMP6, an upstream regulator of hepcidin [6], [7], were also decreased in the OVX mice as compared to those in the sham-operated mice (Figure 1A). Hepcidin is regulated by various factors, including inflammation and cytokines such as interleukin-6 (IL-6) [8]. We examined IL-6 expression in the liver. There were no differences in hepatic IL-6 expression between the sham-operated and OVX mice (data not shown).

Next, we evaluated the expression of FPN in the duodenum. As shown in Figure 1B, the protein level of duodenal FPN was significantly increased at 3 months after ovariectomy in the OVX mice. Similarly, immunohistochemical analysis showed increased FPN expression in the duodenum of OVX mice as compared to that in the duodenum of sham-operated mice (Figures 1C); moreover, changes in FPN localization were observed. In sham-operated mice, duodenal FPN expression was mostly localized around the nuclei in basement villous enterocytes, meanwhile, FPN expression in OVX mice was diffuse in cytoplasmic villous enterocytes (Figure 1D). Serum and liver iron levels were significantly elevated in the OVX mice as compared to those in the sham-operated mice (Figure 1E).

### Effect of Estrogen on Hepcidin Expression in HepG2 and HuH-7 Cells

**Figure 2:**
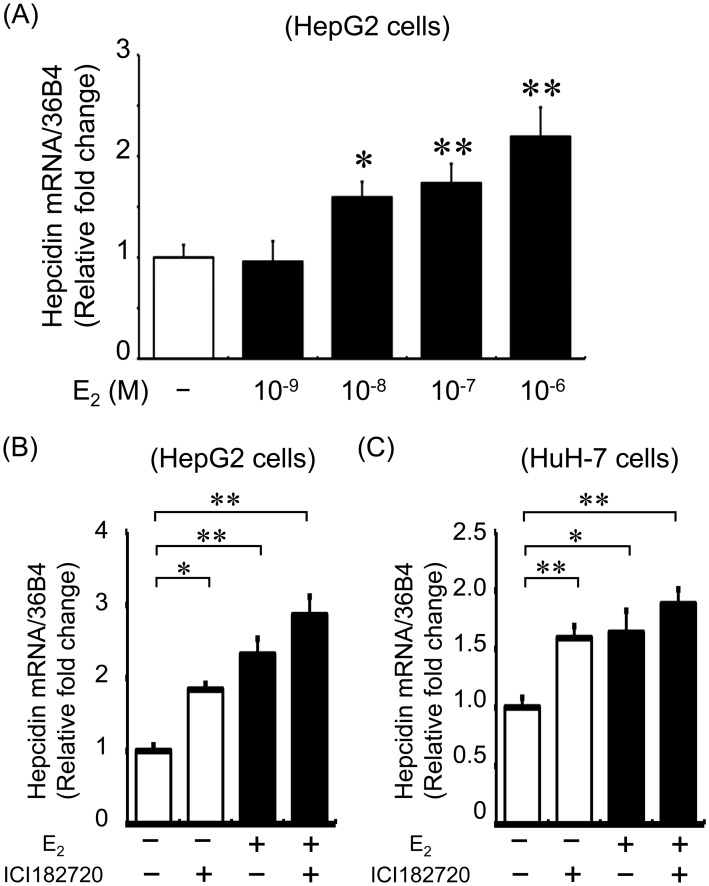
The effect of estrogen on hepcidin expression in HepG2 cells. (A) E_2_ treatment upregulated hepcidin expression in a concentration-dependent manner. HepG2 cells were treated with E_2_ for 24 h. Values are expressed as mean ± SEM; *P<0.05, **P<0.01, *n* = 4–8 in each group. (B) E_2_-induced hepcidin expression was not abolished by the ER inhibitor ICI 182720. HepG2 cells were pretreated with ICI 182720 (1×10^−6^ M) for 1 h before E_2_ treatment. Subsequently, the cells were treated with E_2_ (1×10^−7^ M) or vehicle for 24 h. Values are expressed as mean ± SEM; *P<0.05, **P<0.01, *n* = 4 in each group. (C) E_2_ and ICI 182780 increased hepcidin expression in HuH-7 cells. HuH-7 cells were pretreated with ICI 182720 (1×10^−6^ M) for 1 h before E_2_ treatment. Subsequently, the cells were treated with E_2_ (1×10^−7 ^M) or vehicle for 24 h. Values are expressed as mean ± SEM; *P<0.05, **P<0.01, *n* = 5 in each group.

For further clarification, we examined the effects of estrogen on hepcidin expression *in vitro* using HepG2 cells. As shown in Figure 2A, E_2_ upregulated hepcidin mRNA expression in a concentration-dependent manner. The ER inhibitor ICI 182780 was used to determine whether the effects of estrogen on hepcidin were mediated by an ER-dependent pathway. Interestingly, ICI 182780 co-treatment increased hepcidin expression in both HepG2 (Figure 2B) and HuH-7 cells (Figure 2C).

### E_2_ Induced Hepcidin Expression via GRP30-BMP6-mediated Signaling

**Figure 3:**
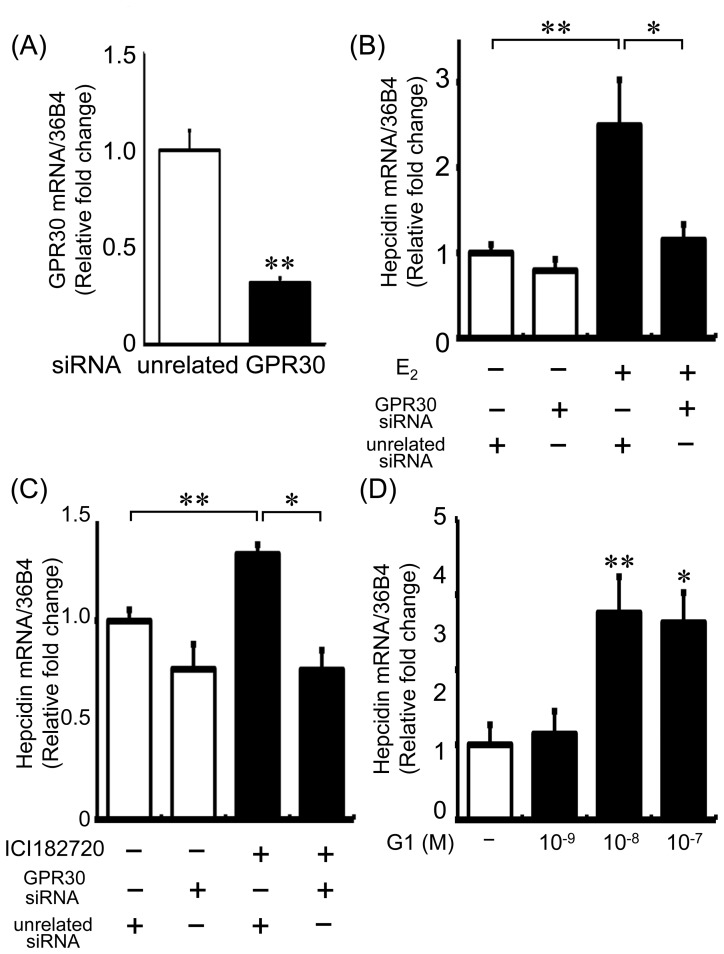
Silencing of GPR30 reduced E_2_-induced hepcidin expression in HepG2 cells. (A) HepG2 cells were transfected with 40 nM GPR30 siRNA. GPR30 mRNA levels were reduced after treatment with GPR30 siRNA; *n* = 4 in each group. (B) Treatment with GPR30 siRNA suppressed E_2_-induced hepcidin upregulation in HepG2 cells. Forty-eight hours after siRNA transfection, HepG2 cells were treated with E_2_ (1×10^−7^ M) or vehicle for 24 h; *n* = 10 in each group. (C) Treatment with GPR30 siRNA decreased ICI 182720-induced hepcidin upregulation in HepG2 cells. Forty-eight hours after siRNA transfection, HepG2 cells were treated with ICI 182720 (1×10^−6^ M) or vehicle for 24 h; *n* = 4 in each group. (D) The effect of the GPR30 antagonist G1 in HepG2 cells. G1 upregulated hepcidin expression in HepG2 cells in a concentration-dependent manner. Values are expressed as mean ± SEM; *P<0.05, **P<0.01, *n* = 4–8 in each group.

**Figure 4:**
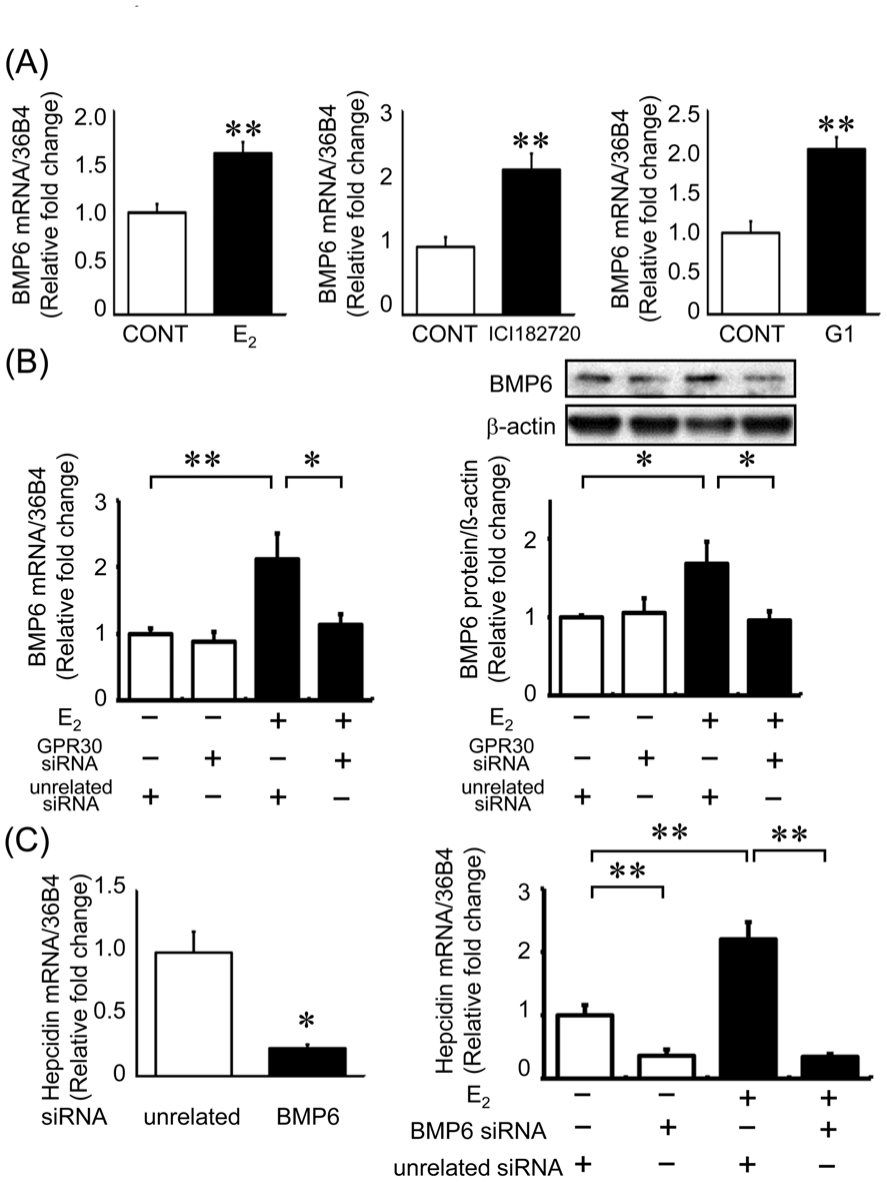
BMP6 signaling is involved in estrogen-induced hepcidin expression via GPR30 in HepG2 cells. (A) Effects of E_2_ (1×10^−7^ M), ICI 182720 (1×10^−6^ M), and G1 (1×10^−8^ M) on BMP6 expression in HepG2 cells. HepG2 cells were incubated for 24 h with each reagent; *n* = 10 in each group. (B) E_2_-induced upregulation of BMP6 expression was inhibited by GPR30 silencing in HepG2 cells. Forty-eight hours after GPR30 siRNA transfection, HepG2 cells were treated with E_2_ (1×10^−7^ M) or vehicle for 24 h; *n* = 8 in each group. (C) BMP6 silencing decreased hepcidin expression in HepG2 cells. Left panel: HepG2 cells transfected with 40 nM BMP6 siRNA. BMP6 mRNA levels were reduced after treatment with BMP6 siRNA. Values are expressed as mean ± SEM; *P<0.05, *n* = 4 in each group. Right panel: Treatment with BMP6 siRNA decreased hepcidin expression in HepG2 cells. Hepcidin downregulation induced by BMP6 silencing was not restored by E_2_ treatment. Values are expressed as mean ± SEM; *P<0.05, **P<0.01, *n* = 8 in each group.

GPR30 has been identified as a membrane receptor for estrogen [9], and several studies have shown that ICI 182780 exerts its effects as an agonist of GPR30 [10]. To determine whether E_2_ augments hepcidin expression through a GPR30-dependent pathway, we performed silencing experiments using siRNA against GPR30. The mRNA levels of GPR30 were decreased by 68% after treatment with GPR30 siRNA as compared to the levels in the control siRNA-treated cells (Figure 3A). GPR30 silencing suppressed E_2_-induced hepcidin expression almost entirely (Figure 3B). Similarly, silencing of GPR30 suppressed ICI 182780-induced hepcidin expression (Figure 3C). Moreover, the GPR30 agonist G1 stimulated hepcidin expression in a concentration-dependent manner (Figure 3D). Similar to hepcidin, BMP6 expression was stimulated by E_2_, ICI 182780, and G1 (Figure 4A). Moreover, E_2_-induced BMP6 upregulation was inhibited by GPR30 silencing in HepG2 cells (Figure 4B). To assess whether E_2_ induced hepcidin expression through BMP6 signaling, we used siRNA against BMP6. BMP6 expression was decreased by 78% after siRNA transfection (Figure 4C left). Silencing of BMP6 itself markedly decreased hepcidin expression, and E_2_-induced hepcidin upregulation was abolished by BMP6 silencing in HepG2 cells (Figure 4C right).

### Effect of E2 or G1 Supplementation on the Repression of Hepcidin and BMP Expression in the Liver and Augmentation of FPN Expression in the Duodenum of OVX Mice

**Figure 5:**
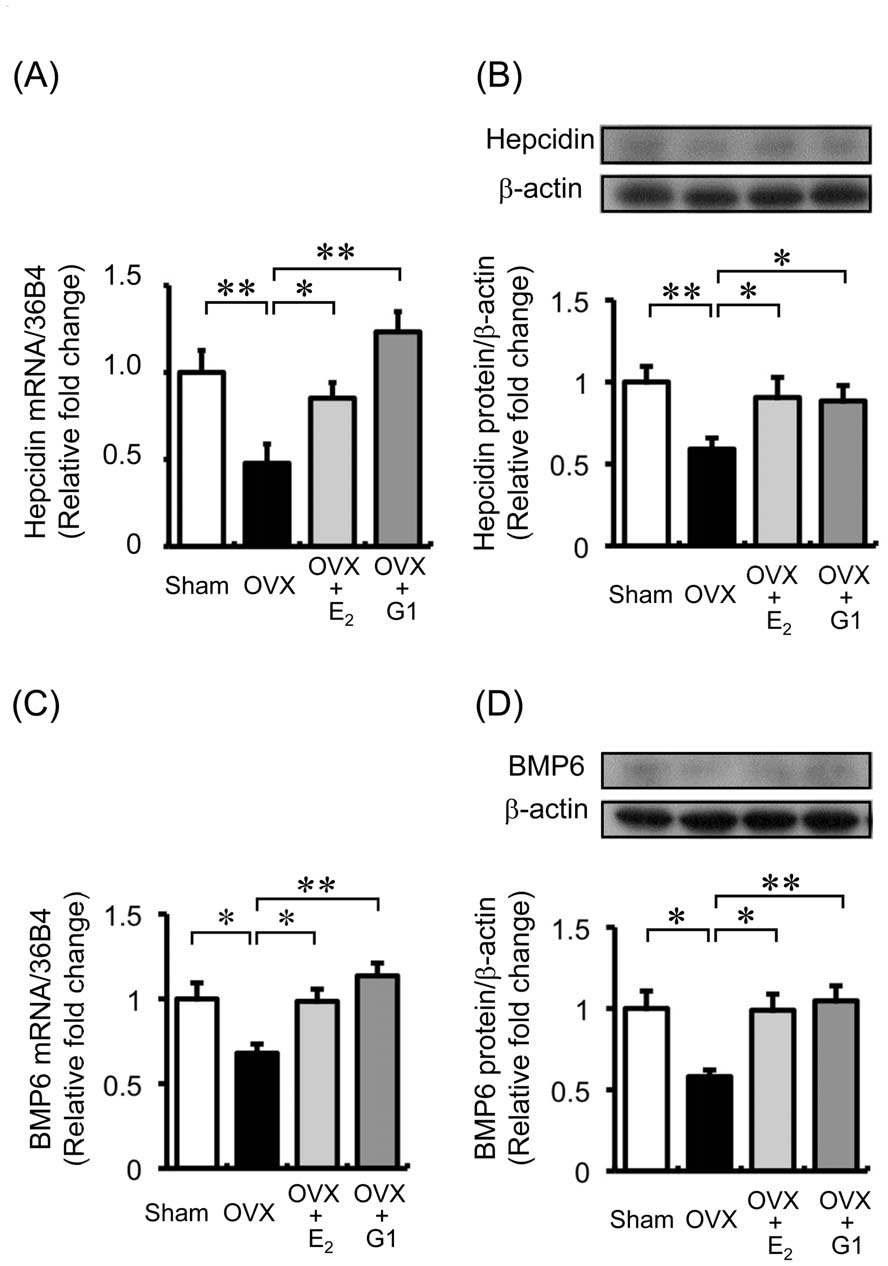
The mRNA and protein expression of hepcidin in the liver. (A) The mRNA expression of hepcidin in the liver of OVX mice was restored by E_2_ or G1 supplementation. Hepcidin expression was decreased in OVX mice (black bar); and its expression was restored by administration of E_2_ (light gray bar) or G1 (dark gray bar). Values are expressed as mean ± SEM; *P<0.05, **P<0.01, *n* = 7 in each group. (B) The protein expression of hepcidin in the liver of OVX mice was restored by E_2_ or G1 supplementation. Hepcidin expression was decreased in the OVX mice (black bar); its expression was restored by administration of E_2_ (light gray bar) or G1 (dark gray bar). Values are expressed as mean ± SEM; *P<0.05, **P<0.01, *n* = 6–8 in each group. The mRNA and protein expression of BMP6 in the liver. (C) The mRNA expression of BMP6 in the livers of OVX mice was recovered by E_2_ or G1 supplementation. BMP6 expression was diminished in OVX mice (black bar); its expression was restored by the administration of E_2_ (light gray bar) or G1 (dark gray bar). Values are expressed as mean ± SEM; *P<0.05, **P<0.01, *n* = 7 in each group. (D) The protein expression of BMP6 in the liver of OVX mice was recovered by E_2_ or G1 supplementation. BMP6 expression was diminished in thr OVX mice (black bar); and its expression was restored by administration of E_2_ (light gray bar) or G1 (dark gray bar). Values are expressed as mean ± SEM; *P<0.05, **P<0.01, *n* = 6–8 in each group. (D).

**Figure 6:**
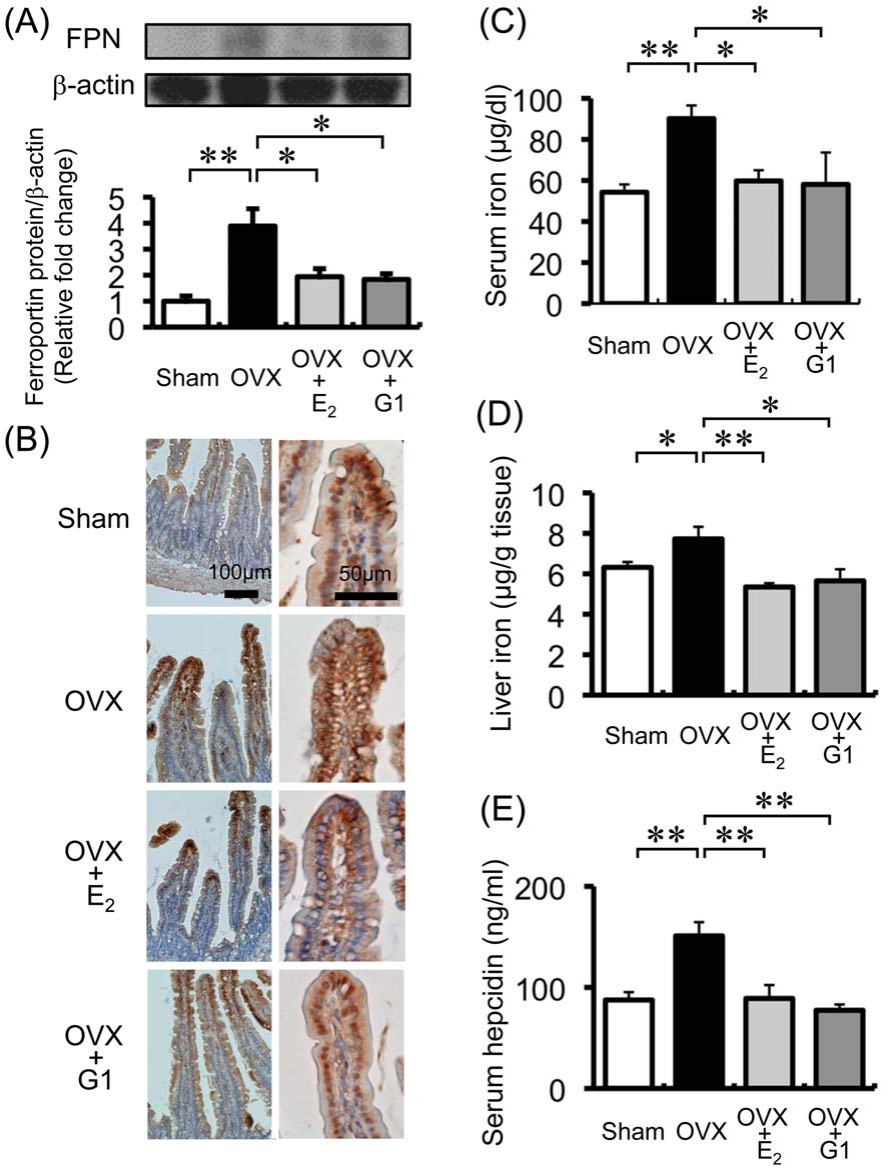
The changes of FPN expression in the duodenum, serum and liver iron, and serum hepcidin. (A) OVX-induced duodenal FPN upregulation was ameliorated in the OVX mice by E_2_ or G1 administration. FPN expression was augmented in OVX mice (black bar); its expression was diminished by treatment with E_2_ (light gray bar) or G1 (dark gray bar). Values are expressed as mean ± SEM; *P<0.05, **P<0.01, *n* = 6 in each group. (B) Immunohistochemical analysis of FPN expression in the duodenal tissue of sham-operated mice, OVX mice, and OVX mice treated with E_2_ or G1. (C) Serum iron levels were increased in the OVX mice (black bar); levels were diminished by the administration of E_2_ (light gray bar) or G1 (dark gray bar). Values are expressed as mean ± SEM; *P<0.05, **P<0.01, *n* = 8–13 in each group. (D) Liver iron concentration in the liver was elevated in OVX mice (black bar); its concentration was reduced by the administration of E_2_ (light gray bar) or G1 (dark gray bar). Values are expressed as mean ± SEM; *P<0.05, **P<0.01, *n* = 8–11 in each group. (E) Serum hepcidin-1 levels were augmented in the OVX mice (black bar) compared to the sham- operated mice (white bar); its levels was reduced by the administration of E_2_ (light gray bar) or G1 (dark gray bar). Values are expressed as mean ± SEM; *P<0.05, **P<0.01, *n* = 8–9 in each group.

We investigated whether reduced hepcidin expression in the OVX mice was restored by E_2_ or G1 supplementation. Administration of either E_2_ or G1 recovered hepcidin and BMP6 expression in the livers of OVX mice (Figure 5). Moreover, the increased FPN protein expression in the duodena of the OVX mice was diminished by E_2_ or G1 treatment (Figure 6A), which was confirmed immunohistochemically (Figure 6B). The expression pattern of FPN in the villous enterocytes of the OVX mice was changed from diffuse to localized by E_2_ or G1 administration (left side of Figure 6B). Serum and liver iron concentrations were increased in the OVX mice, which was restored in the OVX mice with E_2_ and G1 administration (Figure 6C and D). In contrast to liver hepcidin expression, serum hepcidin levels were elevated in the OVX mice compared to the sham-operated mice, while the OVX-induced serum hepcidin increase was reduced by E_2_ or G1 treatment (Figure 6E).

## Discussion

In the present study, we found that hepatic hepcidin expression was downregulated in OVX mice. Consequently, duodenal FPN expression was upregulated. Estrogen played a role in hepcidin expression in a GPR30-BMP6-dependent manner *in vitro*. These findings indicate that estrogen is involved in iron metabolism through the regulation of hepcidin.

Hepcidin, an antimicrobial peptide, is mainly produced and secreted by hepatocytes [11], [12]. FPN, an iron exporter, also plays a pivotal role in intestinal iron absorption [13]. Recently, hepcidin was demonstrated to play a crucial role in iron metabolism by regulating iron absorption in the duodenum via FPN [5]. Indeed, hepcidin-deficient mice showed massive iron overload [14] and increased FPN expression in the duodenum [15]. Thus, the coordinated regulation of hepcidin and FPN is directly linked to duodenal iron absorption. In the present study, OVX mice showed decreased expression of hepatic hepcidin as compared to its expression in sham-operated mice, and E_2_ stimulated hepcidin expression in cultured hepatocytes. These data suggest that estrogen directly participates in the regulation of hepatic hepcidin expression. Indeed, the expression levels of FPN in the duodenum were also augmented in the OVX mice with reduced hepcidin expression, suggesting that accelerated iron absorption and consequent increased iron storage occur in the estrogen-deficient state. In fact, OVX mice showed augmented iron storage in the present study. Estrogen participates in iron metabolism via hepcidin and FPN, which is a new explanation for the increase in iron storage in women during menopause.

Hepcidin expression is regulated by various factors, and BMP6 regulates iron metabolism via hepatic hepcidin [6], [7]. In the present study, BMP6 expression was significantly decreased in the livers of OVX mice as compared to that in the livers of sham-operated mice. In HepG2 cells, E_2_ treatment induced BMP6 expression in addition to hepcidin expression. BMP6 silencing abolished hepcidin expression, and E_2_ failed to upregulate hepcidin expression in HepG2 cells after BMP6 silencing. Moreover, E_2_-induced hepcidin expression as well as BMP6 expression was not suppressed by the ER antagonist ICI 182780. Unexpectedly, ICI 182780 augmented both hepcidin and BMP6 expression. ICI 182780 has been demonstrated to have an agonistic effect on GPR30, a membrane receptor for estrogen [9], [10]. The GPR30 agonist G1 also upregulated the expression of hepcidin and BMP6; conversely, GPR30 silencing suppressed both E_2_- and ICI 182780-induced hepcidin and BMP6 expression. Finally, hepcidin expression in the livers of OVX female mice was recovered by E_2_ and G1 supplementation. These findings suggest that a GPR30-BMP6 pathway, not the classical ER-mediated signaling pathway, is involved in the E_2_-induced expression of hepcidin in the liver.

We also measured serum hepcidin concentrations, which were elevated in the OVX mice compared to sham-operated mice; the increased serum hepcidin levels in the OVX mice were suppressed compared to the levels in sham-operated mice by E_2_ or G1 administration. These results were unexpected and in contrast with the findings of hepatic hepcidin expression. There are several plausible explanations for these data. First, elevated serum hepcidin levels may be a compensatory consequence of upregulated FPN expression in the OVX mice. Berki and colleagues have shown that obese patients or those with diabetes or nonalcoholic steatohepatitis showed reduced hepatic hepcidin expression and increased adipose hepcidin expression compared to lean controls [16], which suggests that serum hepcidin is derived from not only the liver but also from other tissues including adipose. Second, hepcidin expression was reduced in the OVX mice, although serum and liver iron levels were elevated in this study. Some studies have shown that hepatic hepcidin expression is reduced in hepatitis C mice with elevated serum and liver iron content [17], while serum hepcidin levels were increased in patients with hepatitis C [18], which are similar to the results obtained in our study. In addition, serum hepcidin levels correlated with serum iron levels, but not with mRNA levels of hepcidin in the livers of patients with hepatocellular cancer [19]. Thus, the relationship between hepatic hepcidin expression and the serum hepcidin concentration under some clinical conditions remains controversial. Further studies are necessary to clarify this discrepancy between hepatic hepcidin expression and serum hepcidin levels.

In the present study, we demonstrated that estrogen is potentially involved in the regulation of iron absorption via the coordination of hepatic hepcidin and duodenal FPN. However, there is a gender discrepancy between humans and mice with respect to iron metabolism and hepcidin regulation. Body iron content is typically lower in women than in men, whereas several studies on mice have shown that female mice have higher iron content in the liver and spleen than male mice [20], [21]. Females menstruate monthly from adolescence until menopause in humans but not in mice. Regarding the relationship between hepcidin and gender-specific differences in iron storage, hepatic hepcidin expression is higher in female mice than in male mice [20], [21]. In contrast, recent clinical studies have shown that serum hepcidin levels are lower in women than in men [22], and premenopausal women have lower serum hepcidin concentrations than postmenopausal women [23], [24]. Thus, it is difficult to apply our results regarding the effects of estrogen on hepcidin expression to humans.

Although the effects of the relationship between estrogen and hepcidin on iron metabolism remains unclear, testosterone has been demonstrated to participate in iron metabolism via hepcidin. Testosterone administration decreases serum hepcidin concentrations [25]. Women with polycystic ovary syndrome, who have decreased estrogen and increased testosterone levels, also show reduced serum hepcidin levels [26]. In this study, we showed the effects of estrogen on hepcidin expression. Therefore, further study is necessary to determine the mechanisms of action of sex steroid hormones in regulating hepcidin expression as well as iron metabolism.

In conclusion, estrogen regulates iron absorption via hepcidin in the liver. The effects of estrogen on hepcidin expression are exerted through a GPR30-BMP6-dependent mechanism. Estrogen-deficient conditions after ovariectomy resulted in augmented iron absorption in the duodenum because of the downregulation of hepcidin in the liver and contributed to increased body iron storage. Excess iron content is known to cause oxidative stress via the Haber-Weiss reaction [27]. These findings regarding the effects of estrogen on iron metabolism might explain the increase in iron accumulation in estrogen-deficient conditions such as menopausal disorders.

## Materials and Methods

### Materials

We purchased 17ß-estradiol (E_2_), ICI 182780, and the GPR30 agonist G1 from Calbiochem (San Diego, CA), Tocris Bioscience (Ellisville, MO), and Cayman Chemical Company (Ann Arbor, MI), respectively. The following commercially available antibodies were used: anti-FPN (Alpha Diagnostics, San Antonio, TX), anti-BMP6 (Santa Cruz Biotechnology, Santa Cruz, CA), anti-hepcidin (Abcam, Tokyo, Japan), and anti-ß-actin as a loading control (Cell Signaling Technology, Beverly, MA).

### Animal Preparation and Ovariectomy

All experimental procedures were performed in accordance with the guidelines of the Animal Research Committee of the University of Tokushima Graduate School, and protocols were approved by the Institutional Review Board for Animal protection. Six-week-old female C57/BL6J mice were purchased from Nippon CLEA (Tokyo, Japan). The mice were maintained with free access to water and food (Type NMF; Oriental Yeast, Tokyo, Japan). The iron concentration of the food was 0.01%. Before the experiments, mice were randomly divided into 2 groups: ovariectomized (OVX) and sham-operated groups. The bilateral ovariectomy procedure was performed as follows, In brief, the mice were anesthetized by peritoneal injection of 20 mg⋅kg^−1^ pentobarbital. Next, 2 lateral incisions were made in the skin and the muscle layer. The ovaries were extracted through the incision and excised after ligation. Tissue samples were collected 3 months after the operation. In an additional experiment, OVX mice were administered E_2_ or G1 via an osmotic mini-pump during the final month. The concentration and the method of administering E_2_ (20 mg⋅kg^−1^⋅day^−1^) or G1 (0.1 mg⋅kg^−1^⋅day^−1^) were based on previous studies [28], [29].

### Cell Culture

Human hepatoma HepG2 cells and HuH-7 cells were commercially purchased from TAKARA BIO. INC. (Otsu, Japan) and the Japanese Collection of Research Bioresources (Osaka, Japan), respectively. Cells were cultured in phenol red free-DMEM **(**Life Technologies Japan Ltd., Tokyo, Japan), according to the culture protocol. For each experiment, cells at passages 5–8 were used. According to several studies, HepG2 cells exhibit estrogen receptor (ER) expression [30]; however, the expression levels of ERs in HepG2 cells are insufficient to activate classical receptor-mediated effects [31]. Therefore, we examined ER expression in HepG2 cells using quantitative reverse transcriptase polymerase chain reaction (RT-PCR); however, ER mRNA expression was not detected in these cells (data not shown). Therefore, we also used HuH-7 cells, another human hepatoma cell line that has been shown to express ER in several experiments [32]. Before use, E_2_ was dissolved in pure ethanol, and ICI 182720 and G1 were dissolved in DMSO. The final concentration of ethanol and/or DMSO in the cell culture medium was less than 0.5%; moreover, the control groups were added at the same concentration of ethanol and/or DMSO was added to the controls as the vehicle in all experiments. In some experiments, cells were pretreated with ICI 182780 for 1 h before stimulation with E_2_. Cells were grown to confluence and then transferred to serum-free medium before the start of the experiments.

### RNA Extraction and Evaluation of mRNA Expression Levels

RNA extraction, cDNA synthesis, and quantitative RT-PCR methods have been described previously [33], [34]. In brief, tissues or cells were homogenized in TRIzol reagent (Life Technologies Japan Ltd.). RNA extraction and cDNA synthesis were performed according to the manufacturer’s instructions (PrimeScript® RT reagent Kit with gDNA Eraser (Perfect Real Time), TAKARA BIO INC.). Next, quantitative RT-PCR was performed using the iCycler MyiQ2 Real-Time PCR Detection System (BIO-RAD Laboratories Inc., Hercules, CA) with THUNDERBIRD SYBR qPCR Mix (TOYOBO CO., LTD., Osaka, Japan). The following primer sets were used: 5′-GACCAGTGGCTCTGTTTTCC-3′ and 5′-AAAATGCAGATGGGGAAGTG-3′ for human hepcidin (Hamp); 5′-AGCATAACATGGGGCTTCAG-3′ and 5′- CACGTGCACCTCACTCACTT-3′ for human BMP6; 5′-AGGGACAAGCTGAGGCTGTA-3′ and 5′- GCTGAACCTCACATCCGACT-3′ for human GPR30; 5′-CTGCCTGTCTCCTGCTTCTC-3′ and 5′-AGATGCAGATGGGGAAGTTG-3′ for mouse hepcidin (Hamp1); 5′-GAAGGTTGGCTGGAATTTGA-3′ and 5′- ACCTCGCTCACCTTGAAGAA-3′ for mouse BMP6; and 5′-GCTCCAAGCAGATGCAGCA-3′ and 5′-CCGGATGTGAGGCAGCAG-3′ for 36B4 as an internal control [33].

### Small Interfering RNA (siRNA) Experiments

siRNAs targeting human GPR30 and BMP6 and a non-targeting siRNA control sequence were purchased from Sigma-Aldrich (Tokyo, Japan), and transfection was performed as previously reported [33]. Briefly, subconfluent HepG2 cells were transfected for 24 h with 40 nM of each siRNA by using Lipofectamine™ RNAiMAX in Opti-MEM medium (Life Technologies Japan Ltd.) according to the manufacturer’s instruction. Next, the conditioned medium was replaced with fresh serum-free DMEM, after which the cells were incubated for 24 h. For all the experiments, transfected HepG2 cells were used 48 h after transfection. The siRNA sequences were as follows: sense strand 5′-CUGACACCGUCGACCAGGATT-3′ and antisense strand 5′-UCCUGGUCGACGGUGUCGTT-3′ for GPR30 siRNA; sense strand 5′-CAGAAUUCCGCAUCUACAATT-3′ and antisense strand 5′-UUGUAGAUGCGGAAUUCUGTT-3′ for BMP6 siRNA.

### Protein Extraction and Western Blot Analysis

Three months after ovariectomy, the liver and duodenum were removed and stored at −80°C until use. Hepatoma cells were washed with PBS, scraped, and stored at −80°C until further use. Protein preparation and western blotting were performed as previously described [34]. In brief, tissues or cells were homogenized, and proteins were extracted. Extracted proteins were boiled for 5 min in Laemmli sample buffer and then separated using SDS-PAGE. Once the proteins were transferred to a polyvinylidene fluoride (PVDF) membrane, the membrane was blocked for 1 h at room temperature. Next, the membrane was incubated individually with each primary antibody overnight at 4°C, followed by incubation for 1 h with the respective secondary antibody. Immunoreactive bands were detected using a chemiluminescence reagent. Densitometry of the visualized bands was quantified using Image J 1.38× software.

### Immunohistochemistry

Duodenum samples extracted from mice were fixed in 4% paraformaldehyde at 4°C overnight and then embedded in paraffin. Samples were cut into 2-µm sections and deparaffinized. After antigen retrieval with 10 mM citrate buffer at 95°C for 10 min, sections were incubated in primary antibody at 4°C overnight. Staining was visualized by a linked streptavidin-biotin complex assay and a DAB substrate kit (LSAB+ Kit Universal; Dako Japan, Tokyo, Japan). The negative control was incubated without the primary antibody.

### Measurement of Tissue and Serum Iron Concentration

Tissue iron concentration and serum iron levels were measured using an iron assay kit according to the manufacturer’s instructions (Metallo assay, AKJ Global Technology Co., Ltd., Chiba, Japan). In brief, the extracted liver tissues were weighted and homogenized in cell lysis buffer. The crude lysates were further dissolved using an ultrasonic sonicator. Hydrochloric acid (6 N) was added to the samples at a final concentration of 0.05 M; samples were then mixed well and incubated at room temperature for 30 min. Following centrifugation, the supernatants were used for measurements. Tissue iron concentration was corrected using the tissue weight and is expressed as ng⋅g^−1^ tissue.

### Measurement of Serum Hepcidin Levels

Serum mouse hepcidin-1 concentrations were quantitatively analyzed by SELDI-TOF mass-spectrometry as described previously [35]. The assay was performed by Medical Care Proteomics Biotechnology Co.,Ltd. (Kanazawa, Japan).

### Statistical Analysis

Data are presented as mean ± standard error of mean (SEM). An unpaired, 2-tailed Student’s *t*-test was used for comparison between 2 groups. For comparisons between more than 2 groups, the statistical significance of each difference was evaluated by post-hoc test using Dunnett’s method or Tukey-Kramer’s method. Statistical significance was indicated by *P*<0.05.
